# Hand-Book of the Diagnosis and Treatment of Diseases of the Throat, Nose and Naso-Pharynx

**Published:** 1889-03

**Authors:** 


**Hand-Book of the Diagnosis and Treatment of Diseases of the Throat, Nose and Naso-Pharynx.** By CARL SEILER, M. D., Instructor in Laryngology, and Lecturer on Diseases of the Upper Air-Passages, in the University of Pennsylvania, etc., etc. Third edition; thoroughly revised and greatly enlarged. Illustrated with two lithographic plates, containing ten figures, and 101 wood engravings. Cloth, 12mo, pp. xii., 373. Philadelphia: Lea Brothers & Co. 1889.

As Dr. Seiler's book has now reached its third edition, it may be easily claimed that the profession is familiar with it, and has passed a favorable judgment upon it. However, as this edition differs considerably from the previous ones, both in respect to the addition of new material and to the dropping of some of the old, it requires more than the usual notice. Considered as a whole, and as a hand-book, this volume has certain advantages over larger works. Its size and its method of presenting the subject increases its value as a text-book; indeed, we know of none better to put in the hands of students. The publisher's work has been well done. The illustrations are very fine, and many of them, especially those relating to the nose, are better than those in any other book we are familiar with. Every one appreciates how much good illustrations add to the perspicacity of the text, and author and publisher are alike to be commended in the results attained in this volume.

Of the subject-matter of the book we must speak somewhat in detail.

The opening chapters on "The Laryngoscope and the Art of Laryngoscopy" are sufficiently full, and are very clear in their descriptions of the manipulation of the various instruments. We notice the author holds that it is more advantageous to place the reflector on the forehead than over either eye, as we thereby have binocular vision. We agree that binocular vision is to be preferred, but believe it can be just as well obtained with the reflector before one eye or the other, the operator looking with the covered eye through the opening in the mirror, and with the other eye past its edge. This method has the advantage of protecting the eyes, which is important where they are used constantly.

Dr. Seiler prefers the hard-rubber nasal speculum for examining the anterior nasal cavities and, no doubt, in his hands it answers very well. We believe, however, that in a book where so many without experience will obtain their first instructions as to the practice of rhinoscopy, the Bosworth or Elsberg instrument should have been recommended. The hard-rubber speculum requires some care in its introduction, as the author points out, so as not to wound the mucous membrane with its edge, precautions not necessary with the other specula mentioned.

The chapters on "Anatomy and Physiology" are all that could be desired. The latter is new to this work, and will well repay perusal. The function of the larynx is three-fold: *First*, the regulation of respiration; *second*, the protection of the laryngeal cavity and trachea from the introduction of foreign bodies during the act of deglutition; *third*, voice-production or vocalization. The last division is discussed in detail in a most interesting and instructive way, and we advise all those who desire to be well informed on this subject, to become familiar with this chapter. The function of the nose is also of a three-fold nature: The *first* relates to the special sense of smell; the *second* to assisting in voice-production and articulation; and the *third*, and most important, to the preparation of the air prior to its entrance into the lungs. The recognition of the nose as, chiefly, a respiratory organ, has done much to advance the knowledge of its disease and their treatment. The description of this function is clear and satisfactory.

Passing the chapter on "Accessory Instruments," and also the one on "Catching Cold and the Pathology of Mucous Membranes," we come to those treating of disease; and, first, we have to consider the various forms of laryngitis. These are the acute, sub-acute and chronic forms, and also those forms developing in constitutional diseases, as syphilis and phthisis, and that resulting from traumatism. While the limits of a hand-book, such as this, prevents great detail in the consideration of these subjects, we have nothing but praise for the manner in which they have been presented. The student will find here all that is necessary, and obtain very clear ideas as to these different affections. The author states that "a chronic laryngitis is almost always associated with a chronic inflammation of the mucous membrane of the pharynx and naso-pharyngeal cavity, and only by a careful examination into the history of the affection can we determine whether to call it a laryngitis or a pharyngitis." With this opinion we heartily concur.

Functional disorders of the larynx are next considered under the heads of aphonia; aphonia due to cicatricial adhesion; aphonia due to paralysis; unilateral paralysis; and aphonia due to the presence of foreign bodies. The opinion expressed of the previous section applies equally well to this one. Neoplasms of the larynx follow next. Dr. Seiler is rightly conservative in regard to the question, of which we have heard so much lately, as to whether a benign growth may become malignant. He says: "Even when a microscopic examination has determined the nature of a growth, it is often impossible to say whether a tumor is benign or malignant, because neoplasms which formerly were regarded as perfectly harmless, have been known to return after operation, or to change their character from a benign to a malignant form."

The chapter on "Pharyngitis" differs, in a marked degree, from that of the previous editions. Many of the chronic forms have been omitted, as granular and follicular pharyngitis, and pharyngitis sicca. Regarding these omissions, the author says that they "are merely symptomatic expressions of chronic disease of the nose and naso-pharynx, or of gastric irritation, and are, in reality, not entitled to be considered as separate diseases, inasmuch as these heretofore called chronic pharyngites disappear without treatment, with the removal of the cause which produced them." This is quite true. There has been too much refinement and sub-dividing in these diseases, and this step towards simplicity is a stride in advance.

The next chapter deals with "Elongated Uvula and Hypertrophy of the Tonsils." In respect to the latter, Dr. Seiler believes that the best way of treating them is to cut them off as close to the pillars of the fauces as possible. We think he should be more cautious as to a statement of this kind, in a work that will be placed in the hands of students. When such men as Schnitzler, of Vienna; Schmidt, of Frankfort; and others, fear the cutting operation on account of hemorrhage, a more moderate view would seem justifiable. We are not alarmists in regard to this operation, but, nevertheless, believe that the dangers of bleeding following it should be more carefully considered than in this work. Dr. Seiler, rightly we agree, condemns the bistory; he prefers the tonsilitome, except in cases of "ragged" tonsil, when he advises galvano-cautery puncture.

Diseases of the nasal cavities are the next to claim attention. The chapter on "Pathology and Coryza" is very good, and presents the subjects in a concise and clear manner. The next chapter is entitled "Chronic Nasal Catarrh." Dr. Seiler retains the term "Catarrh," though not strictly correct because it is universally used. Two divisions are made of the subject—Hypertrophic and Atrophic Catarrh. The simple chronic catarrh of some authors is not mentioned as a variety distinct from the other two. We think it is better left out, and that Dr. Seiler is right in giving but two varieties. In theory, it is possible to have a chronic inflammation of the nasal cavities without hypertrophy or atrophy, but we believe it is seldom seen in practice.

In the surgical treatment of hypertrophies, the galvano-cautery and the snare are preferred. The various caustics so highly recommended by many, are not considered satisfactory. The galvano-cautery is to be used where the growths are not large and where they are anterior or about the middle of the passages. Where they are large, or posterior, the snare should be used. The Jarvis snare is advised. Perhaps there is none better, but we have used with great satisfaction the Wright instrument. Its chief advantage is that it can be worked with one hand, leaving the other free for any other purpose. The directions for the use of these instruments are so plain that they can hardly be misunderstood. The hard obstructions consist of deviation of the septum, and bony outgrowths. For the correction of the former, the plan of Dr. Steele, of St. Louis, is recommended. This plan has the merit of simplicity, and answers all the requirements of the operation. For the removal of the latter, the burrs, chisel, gouge, and hammer are preferred. We differ from the author in this respect, believing that the saw, as suggested by Bosworth, can be used to better advantage in the majority of cases. This may be a matter of individuality, and, therefore, is not offered as a criticism. As Dr. Seiler justly observes: "We must take into consideration that most operators have a particular fondness for this or that instrument, and prefer to operate with it rather than use any other, if this is possible; probably because they have acquired especial dexterity in its manipulation. It is, therefore, natural that they should praise their pet tool, and obtain results with it which others, with less dexterity in its use, can never hope to arrive at."

The chapter on "Hay-Fever, or Coryza Vaso-Motoria Periodica," the latter being the better term, is new to this edition. It is a good review of the present state of our knowledge concerning this complicated disease. We agree with the author that only palliative treatment is indicated during the attack, as any radical measures will only aggravate it and thus increase the suffering of the patients. The subject of Atrophic Catarrh—that most intractible disease, so discouraging to *both* the physician and patient—is the title of the next chapter. In its treatment, the method of Dr. Bresgen, of Frankfort-on-Main, is advised. This consists, after cleaning the parts, of applying in increasing strength, powders of nitrate of silver and starch, Other methods also receive attention. Patience, perseverance, and the combinations of the various methods, seem to be the sum of our present knowledge regarding the treatment of the disease. The tumors of the nasal passages are also discussed in this. chapter, and the measures of removal given in detail.

The last chapter gives tables of the symptoms of the diseases of the larynx and naso-pharynx. These tables are valuable, besides being convenient. A good index completes the book.

In conclusion, we can but repeat that Dr. Seiler's Hand-book is one of the best we know, especially as a work for students.

The criticisms that have been offered have been as to certain matters of detail. As a whole, its teachings are eminently sound and reliable.

